# Tuberculome myocardique: localisation inhabituelle de la tuberculoseà propos d'une nouvelle observation avec une revue de la littérature

**DOI:** 10.11604/pamj.2016.24.32.9361

**Published:** 2016-05-09

**Authors:** Dalal Lambatten, Sanaa Hammi, Yasmina Rhofir, Jamal Eddine Bourkadi

**Affiliations:** 1 Service de Pneumo-Phtisiologie, Hospital Moulay Youssef, CHU Rabat, Akkari, 10000, Maroc; 2 Faculté de Médecine et de Pharmacie de Rabat, Maroc; 3 Faculté de Médecine et de Pharmacie de Tanger, Maroc

**Keywords:** Tuberculose, cœur, tuberculome, myocarde, imagerie par résonance magnétique, Tuberculosis, heart, tuberculoma, myocardium, magnetic resonance imaging

## Abstract

Nous rapportons l'observation d'un patient de 50 ans présentant une masse tumorale du ventricule gauche évoluant dans un contexte d'altération de l’état général et de fièvre. Cette masse a été objectivée par l’échocardiographie réalisée pour l'exploration d'une cardiomégalie radiologique. L'aspect en imagerie par résonance magnétique était évocateur d'un tuberculome intra myocardique. A travers notre observation, nous proposons une revue de la littérature sur cette localisation inhabituelle de la tuberculose.

## Abstract (en)

We report the case of a 50-year old patient with a left ventricular tumor whose evolution was marked by impaired general condition and fever. This mass was objectified by echocardiography performed to explore a radiological cardiomegaly. The imaging (MRI) appearance was suggestive of an intra-myocardial tuberculoma Based on our observations, we propose a review of the literature on this unusual location of tuberculosis.

## Introduction

La tuberculose est actuellement l'une des principales causes de morbidité et de mortalité par maladies infectieuses. Les localisations cardiaques de la tuberculose sont dominées par les péricardites, mais il existe des atteintes myocardiquesqui posent des difficultés diagnostiques. Ces atteintes peuvent être responsables de mort subite [1, 2]. L'imagerie par résonance magnétique (IRM) cardiaque, examen non-invasif, apporte des éléments déterminants lorsque ce diagnostic est évoqué, comme nous le rapportons dans notre observation.

## Patient et observation

**Figure 1:**
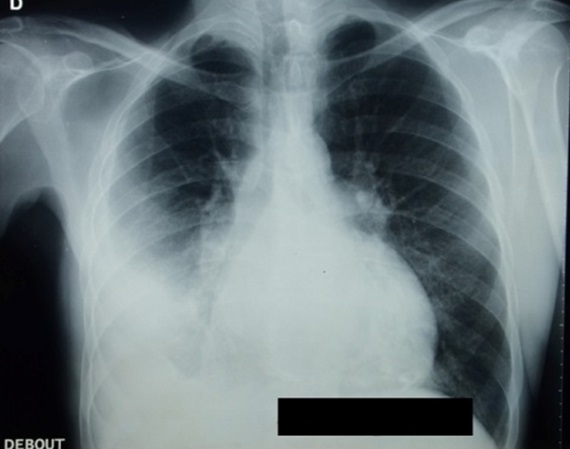
Radiographie thoracique de face

**Figure 2:**
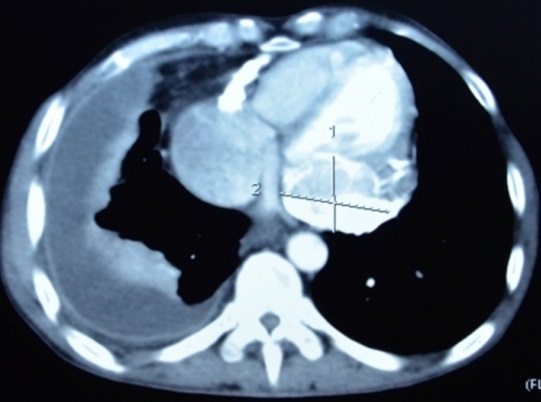
TDM thoracique

**Figure 3:**
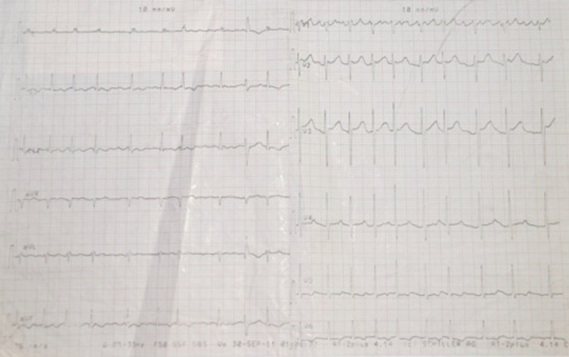
Électrocardiogramme

**Figure 4:**
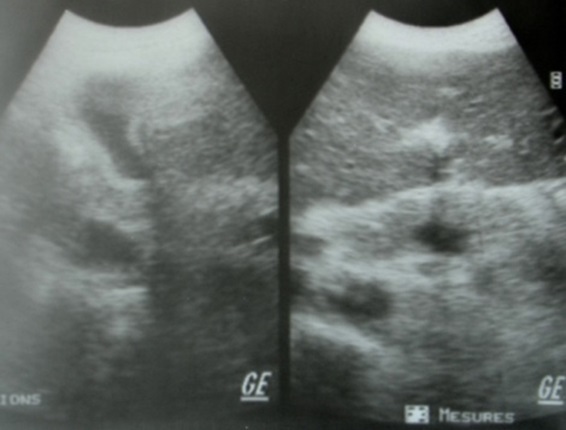
Échocardiographie

**Figure 5:**
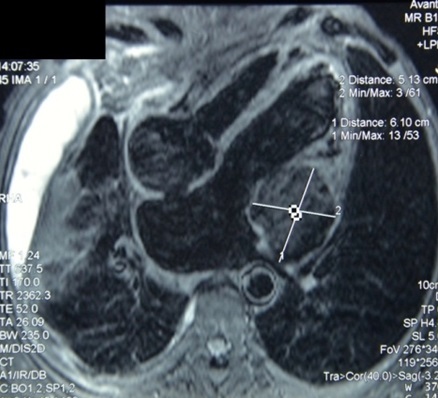
IRM cardiaque

Monsieur M. E, âgé de 50 ans, est admis dans notre formation pour dyspnée. L'anamnèse retrouvait la notion de tabagisme chronique sevré depuis 11 ans, avec notion de frère traité pour tuberculose pulmonaire à microscopie positive il y'a 4 mois. Le patient rapportait 3 mois avant son admission, une toux sèche avec douleur thoracique droite associées à une dyspnée d'aggravation progressive évoluant dans un contexte d'anorexie et d'amaigrissement non chiffrés. A l'examen, le patient était fébrile à 38° avec discrets œdèmes des membres inférieurs et un syndrome d’épanchement liquidien basal droit. L'auscultation cardiaque retrouvait un souffle systolique au foyer mitral avec assourdissement des bruits du cœur. La radiographie thoraciquede face objectivait une pleurésie droite associée à une cardiomégalie modérée avecprojection de calcifications sur l'aire cardiaque (Figure 1). Un complément scannographique thoracique avec injection de produit de contraste objectivait en plus de l’épanchement pleural droit, l'existence d'un processus tissulaire hypodense au niveau du versant externe du ventricule gauche (VG) associé à des calcifications péricardiques (Figure 2). La ponction pleurale retrouvait un liquide jaune citrin, exsudatif (taux de protides à 40 g/l), lymphocytaire à 70%. Deux biopsies pleurales ont été faites et étaient toutefois non concluantes (remaniements inflammatoires non spécifiques). L'intradermo-réaction à la tuberculine était positive à 12 millimètres. La bacilloscopiedans les expectorations ainsi que la sérologie HIV étaient négatives. Le reste du bilan biologique était sans particularité en dehors d'un syndrome inflammatoire. Le bilan cardiologique a comporté, un électrocardiogramme (ECG) qui inscrivait une tachycardie sinusale avec des troubles diffus et concordants de la repolarisation à type d'onde T négative sur l'ensemble des dérivations (Figure 3). Le complément échocardiographique (Figure 4) confirmait l'existence d'une grosse masse de 6 cm×5cm, de structure homogène, contigüe à l'anneau mitral avec dilatation importante de l'oreillette gauche et une hypertension artérielle pulmonaire (pression artérielle pulmonaire systolique à 44 mmHg). L'imagerie par résonnance magnétique (IRM) cardiaque a permis une meilleure caractérisation de la masse en visualisant un processus lésionnel infiltrant de la paroi postéro-externe du VG mesurant 5×6×8,5 cm, de grand axe vertical, discrètement hyperintense en T1, hypointense en T2 se rehaussant progressivement après injection du produit de contraste. Cette masse était entourée par une coque hypointense en séquences T1 et T2 (Figure 5). Cet aspect IRM était évocateur d'un tuberculome à paroi calcifiéeintra myocardique, associé à une péricardite calcifiante non constrictive. L'avis cardio-vasculaire a récusé la biopsie myocardique vu le risque opératoire. Ainsi, le diagnostic de tuberculome myocardique était retenu chez notre patient, devant la fréquence élevée de la tuberculose dans notre pays, la notion de contage tuberculeux, l'altération de l’état général, l'existence d'une péricardite calcifiée, l'intradermo-réaction à la tuberculine positive à 12 mm et l'aspect évocateur à l'IRM myocardique.

Un traitement antituberculeux était entrepris selon les recommandations du programme national de lutte anti-tuberculeuse (PNLAT) comprenant deux mois d'association fixe de 4 antituberculeux (ERIPK4^®^) (ethambutol (E), rifampicine (R), isoniazide (I) et pyrazinamide (PZA)) et 4 mois de bithérapieà base d'isoniazide à la dose de 5 mg/kg/j et de rifampicine à 10 mg/kg/j. Le traitement à visée chirurgicale était différé jusqu’à diminution de la taille de la masse. L’évolution clinique au cours des 6 premières semaines du traitement était marquée par une amélioration de l’état général, reprise pondérale de 2 Kilos, apyrexie et disparition des symptômes respiratoires.

Au quarante cinquième jour du traitement, le patient a présenté un ictère cutanéo-muqueux franc avec un bilan hépatique perturbé justifiantl'arrêt du traitement antituberculeux. Quinze jours après l'arrêt, le patient est décédé dans notre structure. L'autopsie n'a pas été faite suite au refus de la famille.

## Discussion

Les tuberculomes du myocarde sont exceptionnels. Ils ont été décrits dans des séries autopsiques avec une prévalence de l'ordre de 0,02% [3]. De rares cas ont été diagnostiqués chez des patients vivants [4–8]. L'atteinte myocardique peut se faire par voie hématogène, par voie lymphatique, mais souvent il s'agit d'une atteinte par contiguïté à partir d'une péricardite ou d'une adénite tuberculeuse médiastinale [6]. Dans notre cas, l'atteinte par contiguïté reste la plus probable. Les lésions tuberculeuses du myocarde peuvent se présenter sous forme infiltrante diffuse [3] ou sous forme de masse tumorale, cas de notre patient [3, 9]. Les tuberculomes myocardiques semblent être plus fréquemment localisés au niveau des cavités droites [4]. La localisation au niveau des deux cavités a été récemment rapportée [5].

Les manifestations de la tuberculose myocardique sont variables: troubles du rythme [3], blocs auriculo-ventriculaires [10], insuffisance cardiaque congestive [7], insuffisance aortique [5], compression des veines pulmonaires ou de la veine cave supérieure par la masse myocardique [5]. Les complications les plus fréquentes sont la fibrillation atriale et la mort subite [1, 2]. L'IRM cardiaque représente actuellement l'examen d'imagerie le plus important dans la démarche diagnostique [11]. L'aspect caractéristique en séquence T2 montre un signal hypointense central et périphérique avec une ligne mince hyperintense [5]. Dans la plupart des cas publiés, la régression de la masse est quasi-complète dès l'instauration du traitement antituberculeux. Dans le cas des tuberculomes volumineux et compressifs, une résection chirurgicale s'impose et améliore le pronostic [8, 12].

## Conclusion

Bien que rare, le diagnostic de tuberculome cardiaquedoit être discuté, en fonction du contexte, devant une masse intracardiaque en échocardiographie dans les pays à endémie tuberculeuse. Ceux-ci pouvant bénéficier de surveillance et de prise en charge spécifiques.
